# Classification of tobacco leaf diseases based on multi-source remote sensing data

**DOI:** 10.3389/fpls.2026.1727082

**Published:** 2026-02-04

**Authors:** Ke Chen, Jian Guo, Linyi Liu, Xiangzhe Cheng, Shujia Zhuang, Wenjiang Huang, Yingying Dong, Chunming Liu, Kun Huang, Qiuqiang Hou, Shuanglü Shan, Yiwei Guo, Xiaomeng Wang, Tong Zhou, Mei Zhong, Siyu Liang, Lihua Chen, Sijie Luo

**Affiliations:** 1 State Key Laboratory of Remote Sensing and Digital Earth, Aerospace Information Research Institute, Chinese Academy of Sciences, Beijing, China; 2 Honghe Branch of Yunnan Tobacco Company, Yunnan, Mile, China

**Keywords:** continuous wavelet transform, hyperspectral reflectance data, random forest, remote sensing, tobacco leaf

## Abstract

Accurate classification of tobacco leaf diseases is critical for objective disease assessment and management. However, traditional manual observation methods are inherently subjective, and classification approaches based on single-feature extraction often exhibit limited robustness. To address these limitations, this study proposes a tobacco leaf disease classification method based on multi-source data. Hyperspectral reflectance data, leaf area index, and chlorophyll content were selected as the original data sources, and corresponding feature extraction strategies were applied. Continuous wavelet transform was employed to extract discriminative features from hyperspectral reflectance data, while leaf area index and chlorophyll content were normalized using the Z-score method. A random forest algorithm was then used for model training and validation. Experimental results demonstrate that the proposed method achieves an overall classification accuracy of 88.7% with a Kappa coefficient of 0.83, indicating strong classification performance and robustness. These results confirm that the proposed multi-source data-based model provides a reliable and effective approach for tobacco leaf disease classification and offers valuable insights for future research using multi-source remote sensing data.

## 1 Introduction

As an important economic crop, tobacco plays a critical role in supporting the tobacco industry, where leaf quality directly influences production efficiency and market value (Zhang et al., 2023a). In recent years, the demand for timely and accurate monitoring of tobacco growth and disease conditions has increased significantly. Hyperspectral remote sensing (HRS), characterized by its real-time, non-destructive, and efficient acquisition of spectral information, has been widely applied in tobacco cultivation management, including nutrient estimation, growth monitoring, and yield and quality prediction. For nutrient estimation, Zhang et al. applied UAV-based hyperspectral imaging with ensemble learning for nitrogen estimation (Zhang et al., 2023b); Tian et al. combined UAV hyperspectral imaging with advanced spectral preprocessing and machine learning to accurately monitor nicotine content in cigar leaves (Tian et al., 2025); and Wang et al. developed a UAV-based multi-feature fusion model (MLP-GBDT) integrating vegetation indices and texture features to achieve highly accurate chlorophyll content inversion (Wang et al., 2025). These studies collectively demonstrate the effectiveness of HRS in capturing plant physiological information for nutrient monitoring. For growth monitoring, Chatzidimopoulos et al. demonstrated that UAV-based high-resolution hyperspectral imaging enables early detection of downy mildew through real-time monitoring of vegetation indices (e.g., NDVI), facilitating targeted fungicide application (Chatzidimopoulos et al., 2024); Zhang et al., 2026 proposed a UAV-based Recommended Nitrogen Application Index (RNAI) integrating key agronomic traits and vegetation indices to guide precise nitrogen application, thereby improving spatial uniformity and growth management (Zhang et al., 2026). These studies highlight how HRS supports dynamic, field-scale monitoring of tobacco growth. For yield and quality prediction, Li et al. utilized proximal and UAV-based hyperspectral data with selective spectral region modeling and partial least squares regression to predict and map tobacco yield in advance (Li et al., 2025); Zhang et al. employed multi-source data fusion combining UAV hyperspectral features, biophysical, and biochemical parameters with RNN models to improve yield estimation (Zhang et al., 2025a); and Yin et al. showed that VNIR hyperspectral imaging combined with a Diversified Region-based CNN (DR-CNN) can accurately predict moisture, chlorophyll, nitrogen, and sugar content in cigar tobacco leaves across air-curing stages (Yin et al., 2024). These works illustrate the potential of HRS for high-accuracy prediction of both yield and quality in tobacco cultivation.

Beyond growth and nutrient monitoring, timely and accurate disease identification is particularly crucial, as viral infections can spread rapidly and often produce subtle or latent symptoms in their early stages, leading to severe and irreversible damage before visual signs appear. Tobacco leaf diseases—such as mosaic disease and leaf curl disease—are among the major factors that threaten tobacco yield and quality. Their fast transmission through plant contact, agricultural operations, or vector insects can lead to substantial economic losses. Traditional disease identification methods rely heavily on manual inspection, which is labor-intensive, subjective, and inefficient. Although deep learning–based disease identification using RGB images has shown promising results (Lin et al., 2022; Sigit et al., 2022), image acquisition in the field still requires considerable manpower and is difficult to scale. In contrast, remote sensing-based disease identification offers high efficiency and large-area monitoring capability. Prior studies have demonstrated that hyperspectral features can effectively reveal spectral abnormalities in leaves affected by various crop diseases (Wang and Pagay, 2024b; Sawyer et al., 2023; Haagsma et al., 2023; Zhang et al., 2024; Reis Pereira et al., 2024; Bao et al., 2024; Wang et al., 2024a), making HRS a valuable tool for tobacco disease surveillance.

Existing work on tobacco disease identification largely integrates hyperspectral features with machine learning. For instance, Zeng et al. demonstrated that hyperspectral feature–based XGBoost modeling enables highly accurate early detection of tobacco bacterial wilt, while an attention-enhanced visible-light model (Tobacco-AT) offers a competitive, low-cost alternative as disease symptoms progress (Zeng et al., 2023). Chen et al. demonstrated that integrating hyperspectral imaging with machine learning enables accurate, non-destructive identification of PVY- and TMV-infected tobacco leaves, achieving up to 100% accuracy in binary classification and revealing key wavelength indicators of disease severity (Chen et al., 2023). Mao et al. developed a UAV-based hyperspectral–machine learning framework for field-scale detection of Tomato Spotted Wilt Virus in tobacco and demonstrated that red-edge–based feature selection combined with SVM enables highly accurate, non-destructive identification of infected plants (Mao et al., 2025). Fan et al. demonstrated that combining visible–near-infrared hyperspectral imaging with SPA-selected feature bands and a random forest classifier enables highly accurate identification of mold contamination in tobacco leaves (Fan et al., 2024). Chadoulis et al. showed that a 3D-CNN framework using hyperspectral imaging enables accurate and early detection of presymptomatic viral infections in Nicotiana benthamiana by effectively exploiting both spectral and spatial information (Chadoulis et al., 2025). These studies indicate that integrating hyperspectral information with machine learning can significantly improve the robustness and accuracy of crop disease monitoring. However, most existing approaches rely primarily on spectral signatures alone, which may reduce robustness under complex field conditions. This motivates the exploration of multi-source data fusion and advanced feature extraction strategies for more reliable disease identification.

To address these issues, this study proposes a multi-source tobacco disease identification framework that integrates hyperspectral signatures, continuous wavelet transform (CWT) features, and physicochemical parameters. CWT is adopted for hyperspectral feature extraction and dimensionality reduction, motivated by its strong capability in detecting local spectral variations in plant disease studies (Cheng et al., 2023; Yang et al., 2024). The extracted wavelet features are then combined with leaf-area and chlorophyll-related measurements to form a comprehensive feature set. A random forest (RF) classifier is finally employed to identify tobacco disease types.

The major contributions of this study are summarized as follows:

1. We design a multi-source feature learning strategy that integrates hyperspectral, chlorophyll-related, and leaf-area parameters for robust tobacco disease identification.
2. We develop a CWT-based hyperspectral feature extraction pipeline that enhances spectral discriminability while reducing redundancy and noise sensitivity.
3. We demonstrate through extensive experiments that the proposed multi-source + CWT + RF framework achieves high accuracy and strong stability in tobacco disease identification.

## 2 Materials and methods

### 2.1 Study area

**Figure 1:**
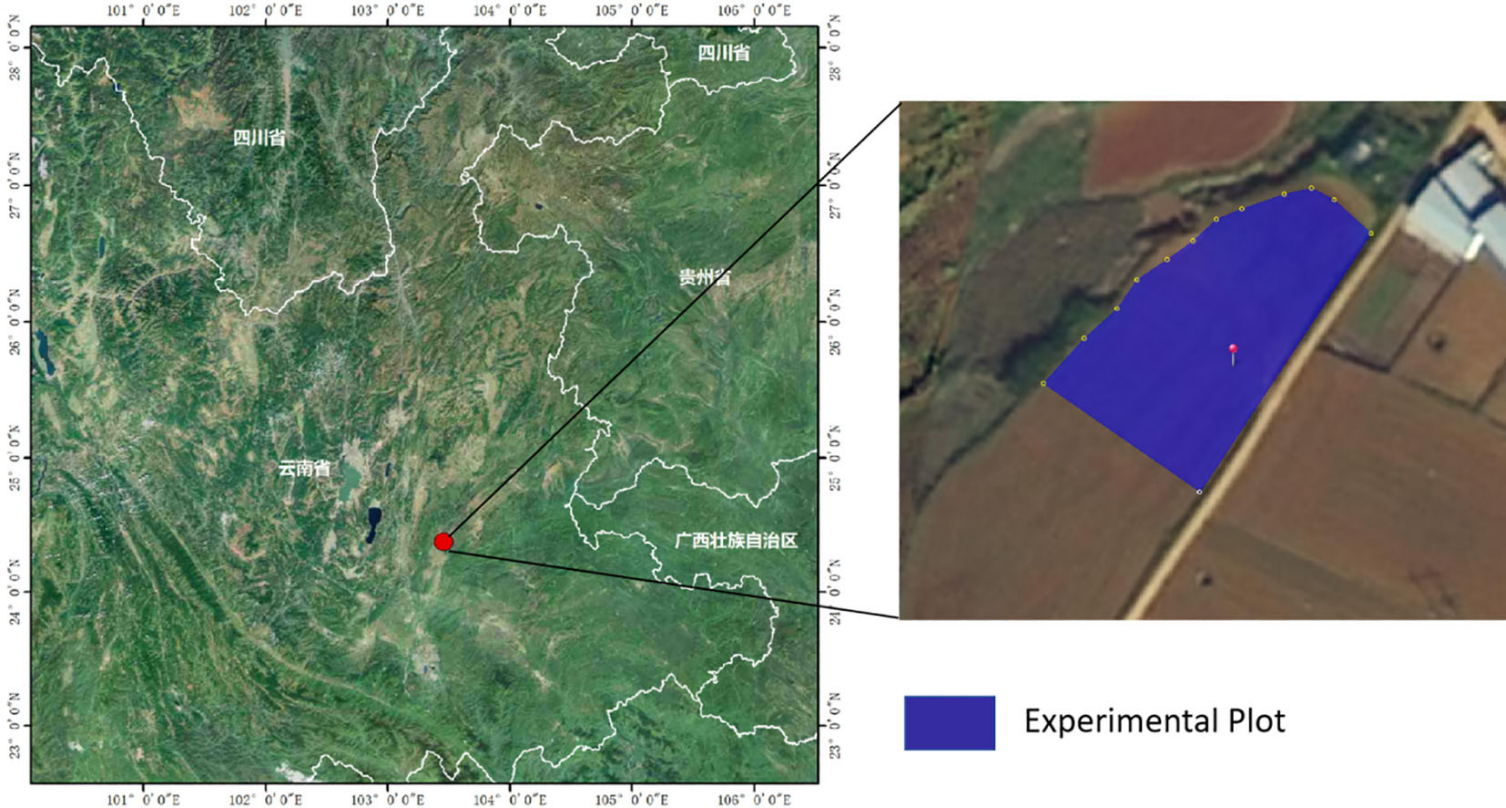
Study area.

The study area is located in the Honghe Tobacco Company’s Olive Slope Flue-cured Tobacco Experimental Demonstration Base in Caihuazhuang, Miyang Town, Mile City, Honghe Prefecture, Yunnan Province, China (24°22′N, 103°27′E), as shown in **Figure 1**. This region belongs to a typical subtropical monsoon climate zone, characterized by abundant sunlight, a long effective growing season, an average annual temperature of 18.8°C, and annual rainfall of 835.4 mm (Mile People’s Government, 2025). Dominant red soil in the study area has good aeration and moderate fertility, providing a favorable growth environment for tobacco. However, the high-temperature, high-humidity conditions also promote the occurrence and development of typical climate-dependent diseases such as tobacco mosaic virus and leaf curl disease. The experiment was conducted from June 26 to June 29, 2024, both in the field and indoors, during a critical growth stage of tobacco, from the seedling to the rooting period. This period also represents the peak incidence and key control stage for tobacco mosaic virus and leaf curl disease. All field measurements were conducted between 10:00 and 14:00 (local time) on clear-sky days to ensure stable illumination conditions. During the surveys, air temperature ranged from 19 to 22°C and relative humidity from 76% to 93%, while the volumetric soil water content averaged 49.4%. Illumination remained stable and adequate throughout the measurement period, and hyperspectral measurements of tobacco canopies were acquired under cloud-free conditions without fog interference, ensuring the reliability of spectral reflectance data.

### 2.2 Data acquisition

A total of 30 tobacco leaf samples were collected from five cultivars (Yunyan 87, Yunyan 105, Yunyan 116, K326, and Yunyan 121) during the field experiment. Each sample was visually inspected and classified into one of three health conditions: healthy (n=10), mosaic disease (n=10), and leaf curl disease (n=10). This class-balanced dataset served as the basis for spectral measurement, leaf area index (LAI) recording, and chlorophyll content acquisition in the following subsections.

#### 2.2.1 Spectral data acquisition

The study used the American FieldSpec Pro FR2500 spectrometer (Analytical Spectral Devices, Inc., Boulder, CO, USA) to acquire hyperspectral data of the tobacco canopy. The instrument has a spectral range of 350 to 2500 nm, with a spectral resolution of 3 nm in the 350 to 1000 nm range and 10 nm in the 1000 to 2500 nm range (Malvern Panalytical, 2025; Vapnik, 1999). The instrument probe has a field of view of 25°. All spectral measurements were conducted under clear weather conditions, with little or no wind, from 10:00 AM to 2:00 PM (Beijing time). During canopy spectral testing, the spectrometer’s spectral probe was oriented vertically downward. Each measurement was taken 10 times. The solar irradiance reflected by the reference white board was measured quasi-synchronously before and after measuring the target canopy reflection. The tobacco canopy spectral reflectance was calculated by the ratio of the measured tobacco canopy radiance to the reference board radiance. The formula is shown in Equation 1:

(1) $$R_{c}=\frac{I_{c}}{I_{r}}\times R_{r}$$

Where $R_{c}$ is the spectral reflectance of tobacco canopy. $I_{c}$ is the radiance (or DN value) of tobacco canopy. $I_{r}$ is the radiance (or DN value) of reference white board. $R_{r}$ is the spectral reflectance of reference white board.

#### 2.2.2 Leaf area index acquisition

The leaf area index was measured in the field experimental plot using a leaf area index meter LI~2200C (LICOR, Inc., Lincoln, NE, USA). The instrument uses a “Fish-eye” optical sensor to measure the transmitted light at five incident angles, achieving observations within a vertical field of view of 148° and a horizontal field of view of 360°. We estimated the leaf area index using a vegetation radiation transmission model with a spectral range of 320nm~490nm.

#### 2.2.3 Chlorophyll content acquisition

**Figure 2:**
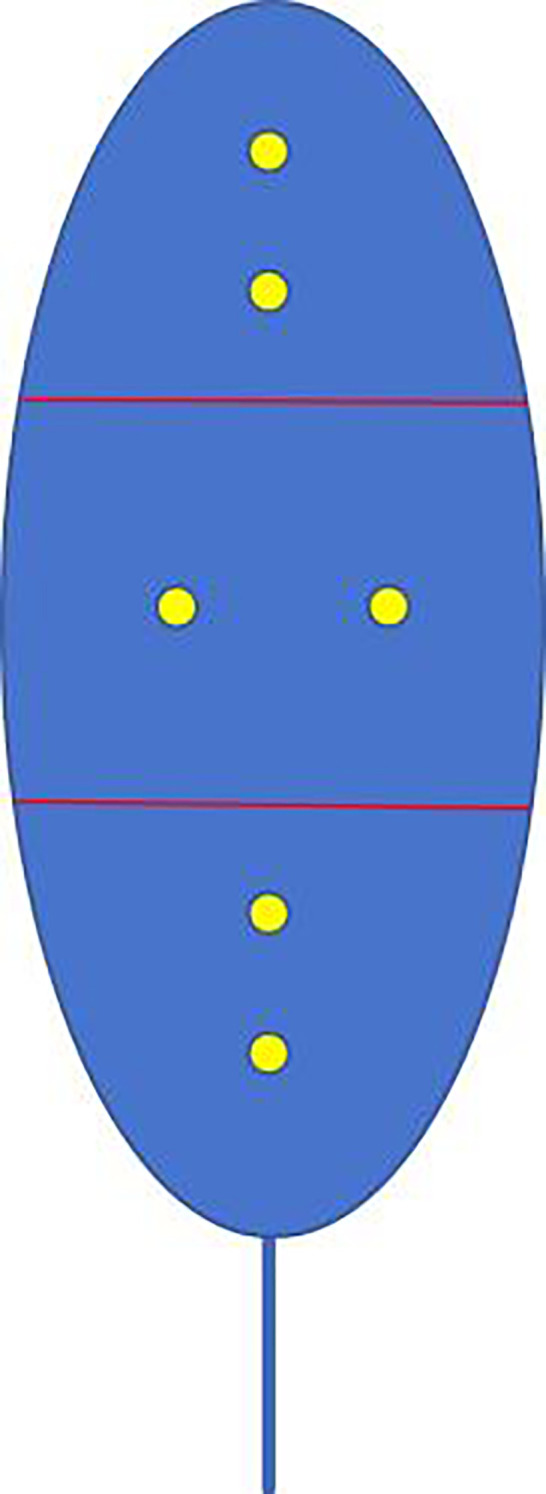
Schematic diagram of chlorophyll content measurement positions on tobacco leaves.

We used a portable chlorophyll meter to acquire the chlorophyll content of tobacco. The data are directly related to the tobacco leaves’ photosynthetic capacity and nutritional status, which is of significant value for the early identification of diseases. The specific method for measuring chlorophyll content in the samples involved collecting two representative and fully expanded leaves from each plant. Each leaf was divided into three sections: upper, middle, and lower as shown in **Figure 2**. Two measurements were taken from the middle of each section, and the average of the six values was considered the chlorophyll content of the leaf. The mean value of these measurements was used as the chlorophyll feature.

**Figure 3:**
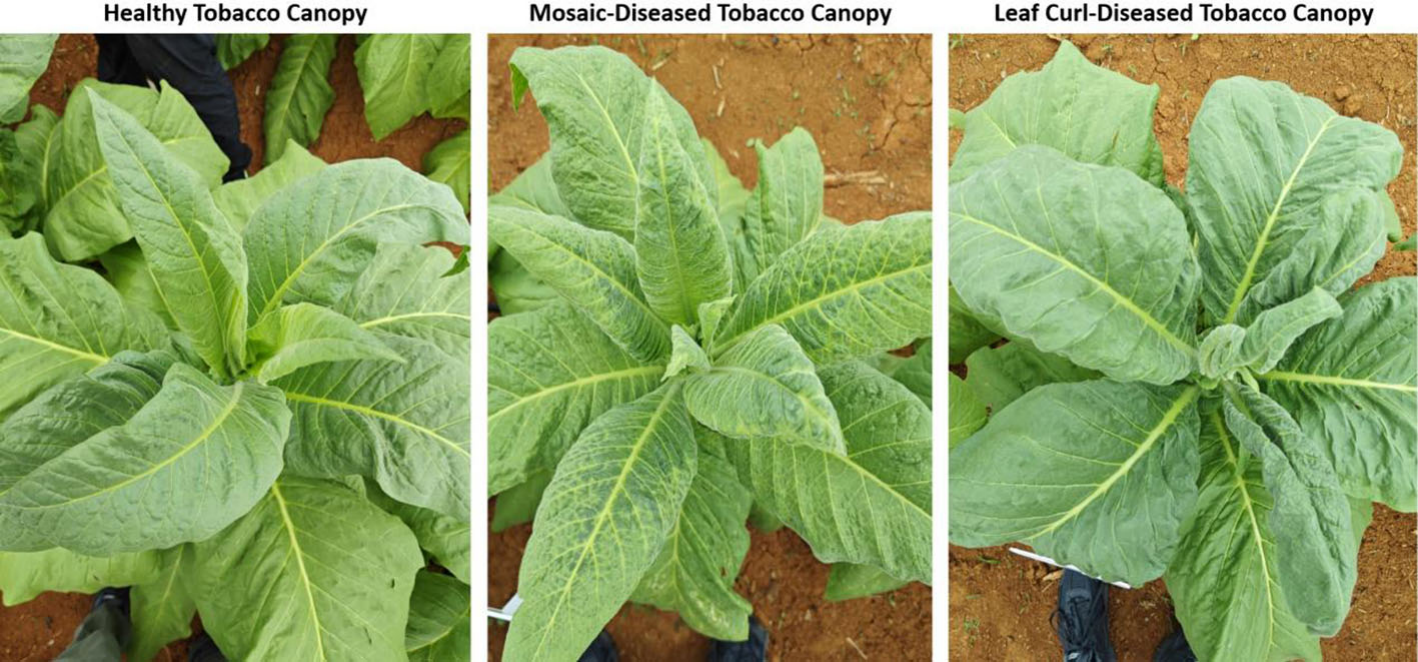
Photographs of healthy tobacco canopy, mosaic-diseased tobacco canopy, and leaf curl-diseased tobacco canopy.

While measuring the above parameters, the tobacco samples were classified into three categories—healthy, mosaic disease, and leaf curl disease—based on the appearance of the leaves and the size of the lesions, as shown in **Figure 3**: (1) Healthy: Leaves with no lesions; (2) Mosaic Disease: The leaf vein tissue becomes light green, with a yellow-green mottled color, and the edges gradually form indentations and curl downward; (3) Leaf Curl Disease: The leaves become more curled, with thickening on the underside, deep green coloration, rolled edges, dark green veins, and the leaves become rigid and brittle with ear-like protrusions on the veins.

### 2.3 Experimental environment

After data collection, the experimental environment including hardware and software configurations was established to ensure reproducibility and reliability of all subsequent analyses and model development. All data processing and modeling were performed using Python 3.9. The computational environment consisted of a laptop with an AMD Ryzen 7 4800U processor (1.80 GHz) and 16 GB RAM.

The following Python libraries and frameworks were employed:

1. pandas for data handling and preprocessing;
2. scikit-learn (sklearn) for feature selection, model construction, and evaluation, including MLPClassifier for BPNN, RandomForestClassifier for RF, and SVC for SVM.

All experiments were conducted in a local environment without GPU acceleration. The environment provided sufficient computational capacity to handle multi-source data features and perform cross-validation for all models efficiently.

### 2.4 Feature extraction and analysis

**Figure 4:**
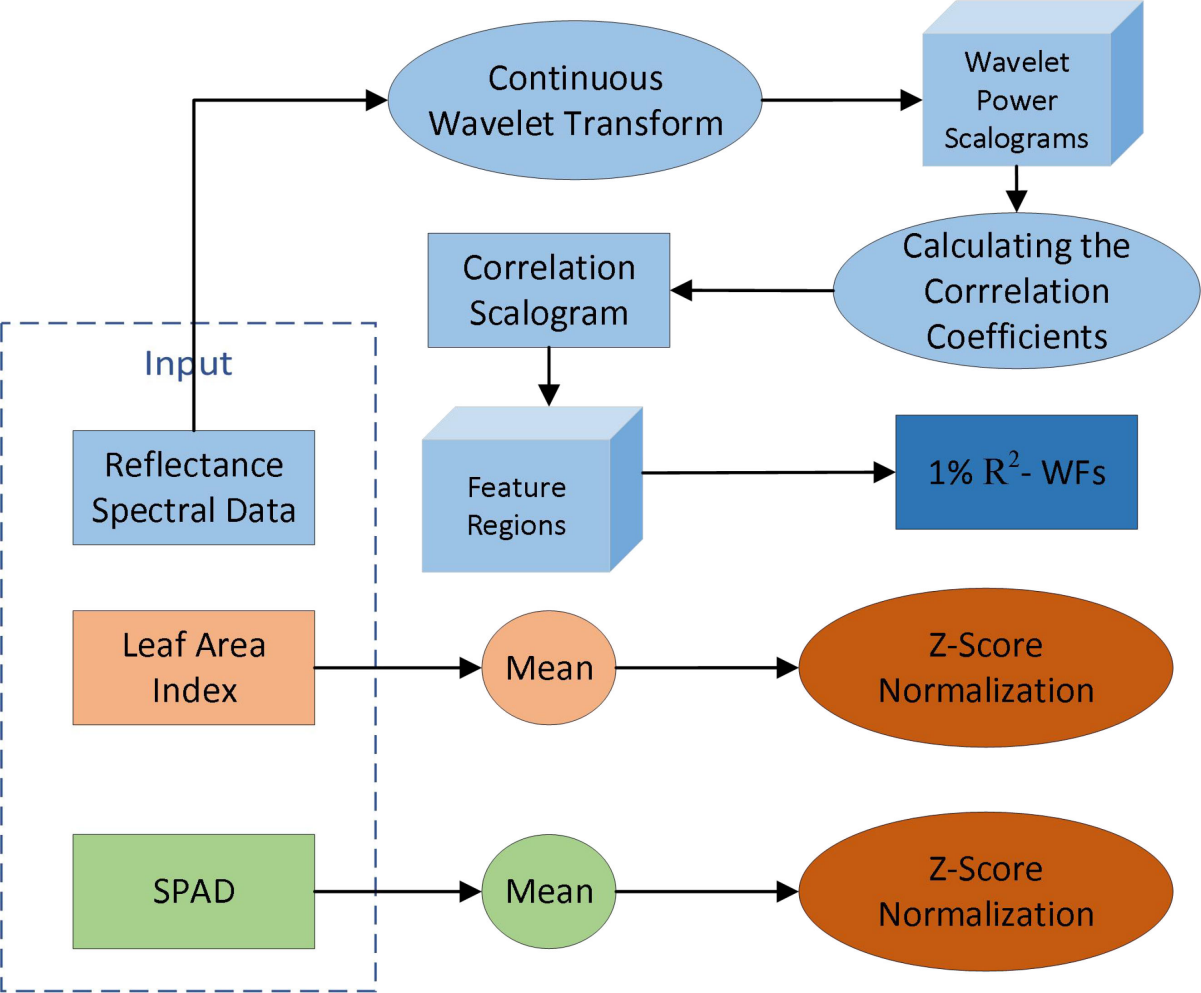
Flowchart of multi-source feature extraction.

This paper combines multi-source data in order to improve the robustness of the model for tobacco leaf disease identification, by extracting features from tobacco hyperspectral reflectance, leaf area index, and chlorophyll relative content data. The feature extraction process is shown in **Figure 4**: the continuous wavelet transform method was used to extract features from the hyperspectral reflectance of tobacco leave; the Z-Score normalization method was used to extract features for chlorophyll content and leaf area index, respectively.

Based on 350~2500nm hyperspectral reflectance data of tobacco leaf samples, we extracted the wavelet features with continuous wavelet transform (CWT). As an effective time-frequency analysis method, CWT is widely used in signal processing, image analysis, feature extraction and other fields. Different from traditional Fourier transform, CWT can capture both time and frequency information of the signal with different scales, particularly suitable for processing non-stationary signals or signals with local variation. CWT expression is shown in Equation 2 as follows:

(2) $$W_{f}\left(a,b\right)=\int_{-\infty }^{+\infty }f\left(\lambda \right)\psi_{a,b}\left(\lambda \right)d\lambda$$

Where f(λ) is the original spectrum, λ=1,2…,m, and m is the number of bands. $W_{f}\left(a,b\right)$ presents the wavelet energy coefficient, $\psi_{a,b}\left(\lambda \right)$ is the mother wavelet basis function used, and its general form is shown in Equation 3 as follows:

(3) $$\psi_{a,b}\left(\lambda \right)=\frac{1}{\sqrt{a}}\psi \left(\frac{\lambda -b}{a}\right)$$

Where *a* is the scale factor of the wavelet width, *b* is the shift factor of the wavelet position.

CWT achieves multi-scale analysis of signals by convolving the signal with a set of mother wavelets with different scales and frequencies. Selecting a suitable mother wavelet is crucial for feature extraction. Common mother wavelets include Mexican Hat wavelet, Morlet wavelet, etc. These wavelets provide a good balance between time domain and frequency domain. The Mexican Hat wavelet (mexh), which has similar absorption characteristics to the vegetation index, was selected as the mother wavelet basis function in this study. In order to simplify the calculation and maintain the accuracy of the continuous wavelet transform method, this study only retained the wavelet power with decomposition scale (*n* = 1, 2, …, 10), referred to as the 1st scale, the 2nd scale, and up to the 10th scale.

Subsequently, this study conducted a correlation analysis between the wavelet energy coefficients extracted by CWT and the tobacco disease grade, and calculated the coefficient of determination (R²). The R² values of the wavelet energy coefficients at different bands and scales constituted a correlation scale diagram, reflecting the sensitivity of each wavelet energy coefficient to tobacco diseases. Based on this, the wavelet energy coefficients with R² in the top 1% were retained and the principal component analysis (PCA) dimensionality reduction was performed. The principal components with a cumulative contribution rate of 95% were retained as the final wavelet features, effectively reducing redundant information.

### 2.5 Model construction

We used the features of multi-source data, including the 1% tobacco hyperspectral features extracted by continuous wavelet transform, the tobacco chlorophyll content (SPAD) features and the tobacco leaf area index (LAI) features extracted by Z-Score correspondingly. Since we adopted multi-source remote sensing data, which constitute a high-dimensional data set, we used the Random Forest (RF) classification method to establish a tobacco leaf pest and disease recognition model in order to reduce the risk of overfitting. We also used support vector machine (SVM) (Breiman, 2001; Smola and Schölkopf, 2004; Karatzoglou et al., 2006) and back propagation neural network (BPNN) (Fei et al., 2023) as comparative classification methods to verify the performance of the random forest classification algorithm.

**Figure 5:**
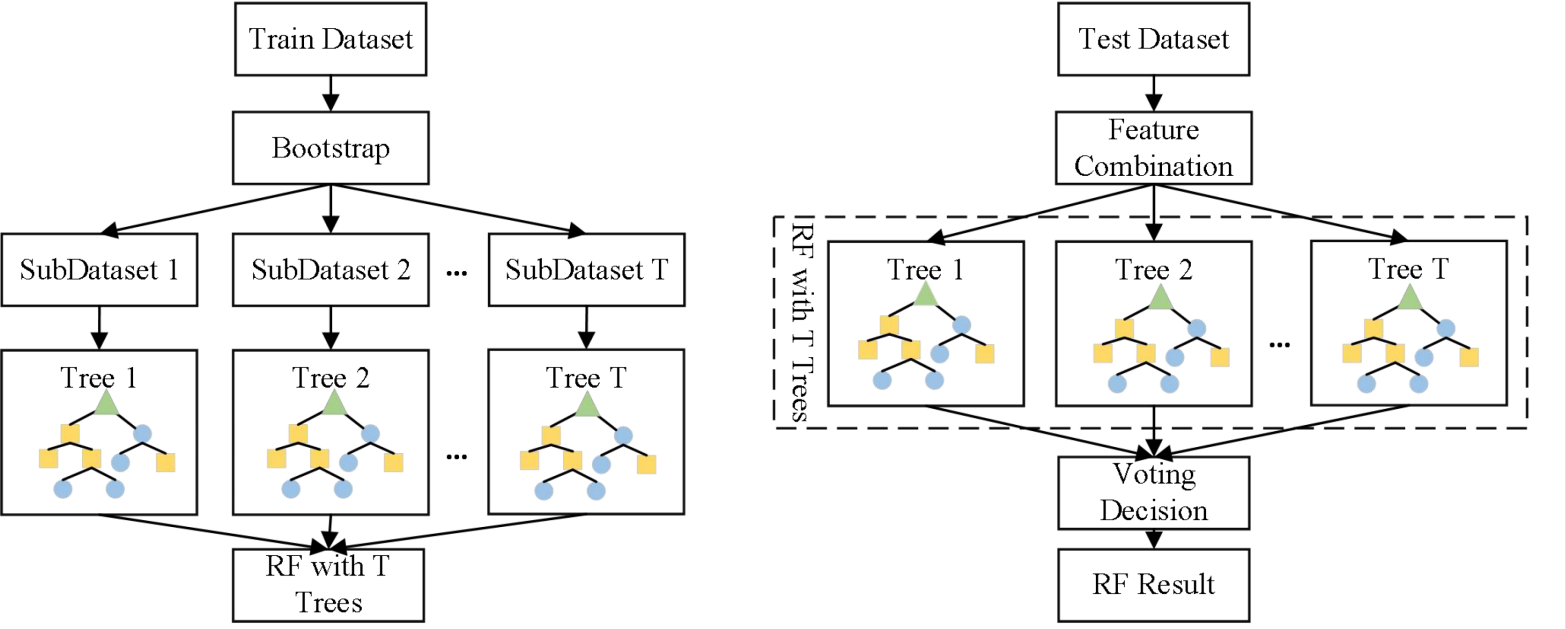
Process of random forest classification.

Random Forest algorithm is a classification model based on ensemble learning (Boulent et al., 2019). It generates multiple sample sets through random sampling with replacement, then constructs a classification tree using the complete split method, and finally averages the classification results of all single binary decision trees to obtain the final classification result, as shown in **Figure 5**. The specific random forest method is as follows:

1. Input sample sets $D=\left\{\left(x_{1},y_{1}\right),\left(x_{2},y_{2}\right),\ldots ,\left(x_{n},y_{n}\right)\right\}$;
2. Use the Bootstrap method to sample samples and obtain sample sets containing m samples $D^{\prime }=\left\{\left(x_{i},y_{i}\right)\right\},\;i\in \left\{1,2,\ldots ,m\right\}$
3. Use the sampled set $D^{\prime }$ to train the decision tree model $G_{t}\left(x\right),t\in \left\{1,2,\ldots ,T\right\}$.
4. The category with the most votes from T decision tree models is the final category.

### 2.6 Accuracy assessment

The samples were divided into a training set and a validation set in a ratio of 7:3 applied within each class, so that 70% of the samples from each class were used for training and the remaining 30% for testing. A hold-out validation strategy was adopted, and the Random Forest classifier was trained on the training portion. All performance metrics were calculated using the testing subset to ensure unbiased accuracy evaluation.

In order to quantitatively evaluate the accuracy and stability of the proposed classification method, the accuracy of the model was evaluated by calculating the overall accuracy (OA), precision, recall, and F1-score. The OA represents the proportion of correctly classified samples among all samples across all classes and is calculated as follows:

(4) $$OA=\frac{\sum_{i=1}^{C}TP_{i}}{\sum_{i=1}^{C}N_{i}}=\frac{Total\;number\;of\;correctly\;classified\;samples}{Total\;number\;of\;samples}$$

Where ***C*** is the total number of classes, *TP_i_* (True Positive) is the number of samples that truly belong to class *i* and are correctly predicted as class *i*; and *N_i_* is the total number of samples in class *i*. Overall Accuracy (OA) as shown in Equation 4 is calculated as the ratio of correctly classified samples to the total number of samples across all classes.

Precision, Recall and F1-score for class *i* is shown in Equations 5–7:

(5) $${\text{Precision}}_{i}=\frac{TP_{i}}{TP_{i}+FP_{i}}$$

(6) $$Recall_{i}=\frac{TP_{i}}{TP_{i}+FN_{i}}$$

(7) $${\text{F}1‐\text{score}}_{i}=2\times \frac{{\text{Precision}}_{i}\times Recall_{i}}{{\text{Precision}}_{i}+Recall_{i}}$$

Where $FN_{i}$ (False Negative) is the number of samples that truly belong to class *i* but are misclassified as a non-*i* class; $FP_{i}$ (False Positive) is the number of samples that truly belong to a non-*i* class but are misclassified as class *i*.

In addition, this paper used the Kappa coefficient to test the classification consistency of the model. The calculation formula is as shown in Equation 8 as follows:

(8) $$K=\frac{OA-p_{e}}{1-p_{e}}$$

$p_{e}$ is composed of a confusion matrix, which is obtained by taking the inner product of the number of real samples and the number of predicted samples of each class. $p_{e}$ can be expressed as Equation 9:

(9) $$p_{e}=\frac{\sum_{i=1}^{N}{\hat{y}}_{i}y_{i}}{N\times N}$$

Where *N* represents the number of sample types, ${\hat{y}}_{i}$ represents the number of samples predicted to be of the *i*-th class, and $y_{i}$ represents the number of true samples of the *i*-th class.

## 3 Results

### 3.1 Spectral feature extraction of tobacco diseases based on hyperspectral data

**Figure 6:**
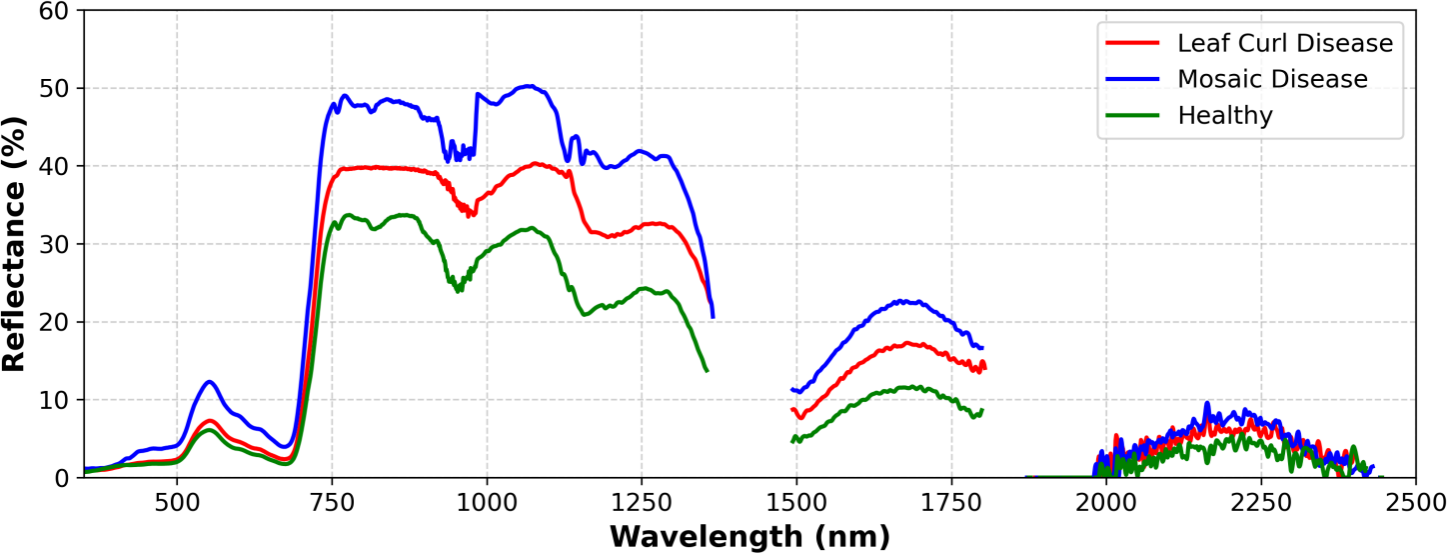
Spectral reflectance curve of Yunyan 87 samples.

**Figure 6** shows the spectral reflectance curves of healthy tobacco leaves, mosaic tobacco leaves and leaf curl tobacco leaves. The spectral reflectance curves of healthy and diseased tobacco leaves show similar overall trends. However, there are obvious differences in the spectral reflectance between samples with different diseases. Between 500nm-1500nm, the reflectance of diseased tobacco leaves is significantly higher than that of healthy tobacco leaves.

**Figure 7:**
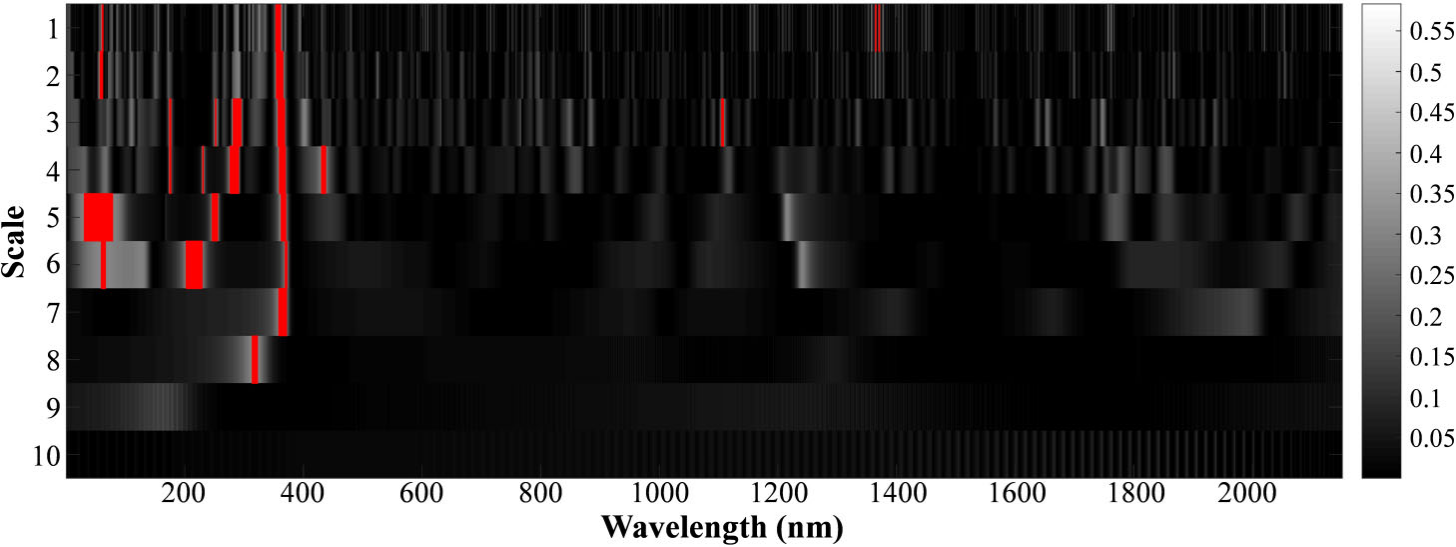
Continuous wavelet transform correlation scale maps.

**Figure 7** plots the correlation scale diagram between tobacco disease levels and leaf spectral reflectance based on CWT. In this study, the R^2^ value ranges from 0 to 0.575. To retain the most informative wavelet features and suppress weakly correlated components, the wavelet features are ranked according to their R^2^ values, and the extracted R^2^ features in the top 1% are selected, which is highlighted with red in **Figure 7**. The results show that the extracted wavelet features are mainly concentrated in the visible light range of 400-760nm and the 1st to 8th scales, with more distribution in the 3rd and 4th scales. To further reduce feature redundancy, principal component analysis (PCA) is applied to the selected wavelet features. When the cumulative contribution rate reaches 95%, six principal components are retained, indicating that most of the informative variance is preserved after dimensionality reduction. In addition, there is a significant correlation between the six selected features and tobacco disease levels (P-value< 0.01). Thus, it can be considered that the extracted wavelet features have high sensitivity to tobacco diseases.

**Figure 8:**
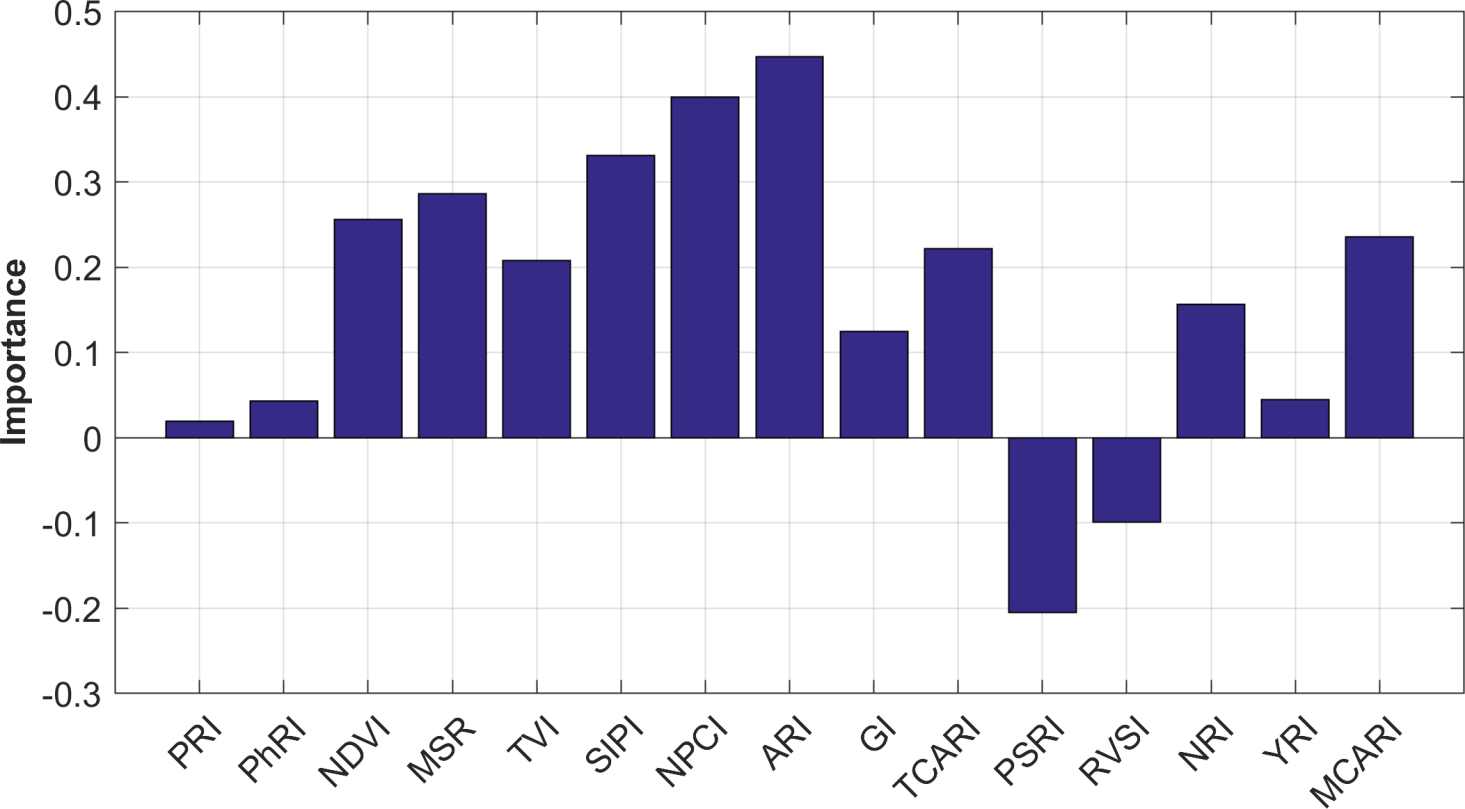
Feature importance evaluation results based on random forest.

In terms of vegetation indices, this study selected the indices listed in **Table 1** and employed the Random Forest feature importance evaluation method to assess the ability of each index to distinguish between healthy tobacco samples and disease-infected tobacco samples. The results are shown in **Figure 8**. As illustrated, NDVI, MSR, SIPI, NPCI, and ARI rank higher in feature importance, indicating that these vegetation indices exhibit strong sensitivity in distinguishing among healthy tobacco canopies, mosaic-diseased tobacco canopies, and leaf curl-diseased tobacco canopies.

**Table 1:** Vegetation indices used in this study.

Vegetation index	Equation
PRI	$\left(R_{570.}-R_{531}\right)/\left(R_{570.}+R_{531}\right)$
PhRI	$\left(R_{550.}-R_{531}\right)/\left(R_{550.}+R_{531}\right)$
NDVI	$\left(R_{830.}-R_{675}\right)/\left(R_{830.}+R_{675}\right)$
MSR	$\left(R_{800.}/R_{670}-1\right)/\sqrt{R_{800.}/R_{670}+1}$
TVI	$0.5(120(R_{750.}-R_{550})-200\left(R_{670}-R_{550}\right))$
SIPI	$\left(R_{800}-R_{445}\right)/\left(R_{800.}-R_{680}\right)$
NPCI	$\left(R_{680}-R_{430}\right)/\left(R_{600.}+R_{430}\right)$
ARI	${\left(R_{550}\right)}^{-1}-{\left(R_{700}\right)}^{-1}$
GI	$R_{554}/R_{667}$
TCARI	$3\left(\left(R_{700}-R_{675}\right)-0.2\frac{\left(R_{700}-R_{500}\right)}{\left(R_{700}/R_{670}\right)}\right)$
PSRI	$\left(R_{680}-R_{500}\right)/R_{750}$
RVSI	$\left(R_{712}+R_{752}\right)/2-R_{732}$
NRI	$\left(R_{570}-R_{670}\right)/\left(R_{570.}+R_{670}\right)$
YRI	$\left(R_{730}-R_{419}\right)/\left(R_{730.}+R_{419}\right)+0.5R_{736}$
MCARI	$\left(\left(R_{701}-R_{671}\right)-0.2\left(R_{701}-R_{549}\right)\right)/\left(R_{701.}/R_{671}\right)$

### 3.2 Evaluating tobacco diseases classification model

Based on the selected wavelet features, vegetation indices, chlorophyll content, and leaf area index, RF, SVM, and BPNN were used to construct classification models for distinguishing healthy tobacco leaves, mosaic-diseased tobacco leaves, and leaf curl-diseased tobacco leaves. Specifically, the BPNN (MLPClassifier) was configured with one hidden layer of 50 neurons, solver=sgd, learning_rate_init=0.01, and max_iter=1000; the SVM used a linear kernel; and the Random Forest (RF) model was configured with 100 estimators. The validation results are shown in **Table 2**. The overall accuracy (OA) of the RF algorithm was 14.3% higher than that of SVM and BPNN. The Kappa coefficient of the RF, SVM and BPNN was 0.83, 0.65, and 0.66, respectively. The experimental results show that the multi-source data features used in this paper perform well in identifying tobacco leaf diseases, proving the feasibility of combining CWT with machine learning for tobacco leaf disease identification. The model combining 1% R^2^-WFs and RF performed well among all models, with OA of 88.9% and a kappa coefficient of about 27.7% higher than that of SVM and BPNN.

**Table 2:** Comparison of classification model results based on classification algorithms.

Classification algorithms	Disease categories	Precision	Recall	F1-score	OA (%)	Kappa
RF	Healthy	1.00	1.00	1.00	88.9	0.83
	Leaf Curl	1.00	0.67	0.80		
	Mosaic	0.75	1.00	0.86		
BPNN	Healthy	0,75	1.00	0.86	77.8	0.67
	Leaf Curl	1.00	0.33	0.50		
	Mosaic	0.75	1.00	0.86		
SVM	Healthy	0.60	1.00	0.75	77.8	0.67
	Leaf Curl	1.00	0.33	0.50		
	Mosaic	1.00	1.00	0.80		

To provide a more detailed view of the classification performance for each disease category, the confusion matrices of the three classifiers are presented in **Table 3**. As shown, the RF classifier correctly classified all healthy and leaf curl samples, with only one Mosaic leaf misclassified, highlighting its superior robustness. In contrast, SVM misclassified one healthy and two Mosaic leaves, while BPNN misclassified one sample in each class, indicating relatively lower performance in handling complex, multi-source features. These confusion matrices further confirm that the RF model is the most reliable approach for accurate tobacco leaf disease identification.

**Table 3:** Confusion matrices of BPNN, SVM, and RF models for tobacco leaf disease classification.

Actual \ Predicted	Healthy	Leaf curl	Mosaic
(a) RF			
Healthy	3	0	0
Leaf Curl	0	2	1
Mosaic	0	0	3
(b) BPNN			
Healthy	3	0	0
Leaf Curl	1	1	1
Mosaic	0	0	3
(c) SVM			
Healthy	3	0	0
Leaf Curl	2	1	0
Mosaic	0	0	3

The superior performance of RF can be attributed to its ensemble learning mechanism, which reduces overfitting and effectively handles high-dimensional, multi-source features. In contrast, SVM and BPNN are more sensitive to feature scaling and may not fully capture complex interactions among features. A simple statistical validation comparing OA and Kappa across the three algorithms further confirms this finding, demonstrating that RF provides a robust and reliable approach for tobacco leaf disease classification.

## 4 Discussion

Tobacco is an important economic crop in China, and the prevention and control of pests and diseases remain key challenges in the process of cultivation. Statistics show that tobacco yield losses caused by pests and diseases account for approximately 10%–15% of the total output value. Accurate and timely identification of tobacco diseases is thus of great importance to reducing economic losses and ensuring crop quality. The spectral reflectance analysis results (**Figure 6**) in this study indicate that diseased tobacco leaves exhibit significantly higher reflectance than healthy leaves in the 500–1500 nm range, primarily due to the reduction in chlorophyll and physiological degradation in infected tissues. This validates the feasibility of using hyperspectral reflectance to discriminate disease severity in tobacco leaves.

Continuous wavelet transform was employed to extract detailed spectral features from hyperspectral data. As shown in **Figure 7**, the wavelet features with strong correlation to disease grades were mainly concentrated in the visible range (400–760 nm) and lower decomposition scales (1st to 8th), especially at the 3rd and 4th scales. These scales are more responsive to subtle pigment and cell structure changes caused by viral infections. Furthermore, principal component analysis was applied to reduce data redundancy while retaining 95% of the cumulative contribution, resulting in six sensitive wavelet features significantly correlated with disease level (P< 0.01). This demonstrates that wavelet-transformed hyperspectral features can effectively reflect disease-induced spectral variations.

In addition to spectral data, the study also incorporated vegetation indices and physiological indicators such as chlorophyll content and leaf area index. Random Forest feature importance ranking (**Figure 8**) revealed that NDVI, MSR, SIPI, NPCI, and ARI were highly sensitive to disease discrimination, aligning with their known association with plant vigor and pigment content. The integration of multiple data sources improved the robustness of disease feature representation and reduced the influence of noise in any single data modality.

Classification performance analysis (**Table 2**) showed that the RF model achieved the highest overall accuracy (OA = 88.9%) and Kappa coefficient (0.83), outperforming SVM and BPNN by 14.3% and over 27% in Kappa, respectively. RF also maintained a good balance between precision and recall across all disease categories, especially for healthy and mosaic-infected leaves. The use of ensemble learning in RF likely contributed to better generalization and resistance to overfitting in high-dimensional data settings. By contrast, SVM and BPNN exhibited unstable classification for healthy leaves, likely due to class imbalance and insufficient feature separation in their internal representations. Given the limited sample size in this study, classical machine learning models remain more suitable and interpretable, as they are less prone to overfitting and can achieve stable performance under small-sample conditions.

Nevertheless, several limitations remain. Although the RF model demonstrated good classification performance under controlled experimental conditions, its generalization capability in complex field environments is still subject to variability, especially under differing illumination, background interference, or early asymptomatic disease stages. Furthermore, the current model relies on manually extracted features and shallow classifiers, which may limit its scalability and adaptability to unseen disease types or growth stages.

Therefore, future work will focus on expanding the tobacco disease dataset under diverse environmental conditions and growth stages. With sufficiently large-scale data, end-to-end deep learning frameworks, such as convolutional or sequence-aware models, may be explored to automatically learn hierarchical representations from raw hyperspectral or RGB data. However, it is worth noting that deep learning models such as CNN or CNN-LSTM typically require large amounts of labeled data and may suffer from overfitting or limited interpretability when training samples are scarce. In contrast, the proposed method is more suitable for small-sample agricultural disease scenarios, offering better robustness and interpretability under limited data conditions. These approaches are expected to further enhance model generalization and robustness in large-scale tobacco disease monitoring scenarios.

## 5 Conclusions

This study is guided by the principle that healthy tobacco leaves have higher chlorophyll content and significantly lower hyperspectral reflectance compared to diseased leaves. We select multi-source remote sensing data, including tobacco hyperspectral data, leaf area index and chlorophyll content as feature data for tobacco disease recognition. Different feature extraction algorithms were applied to these three types of data: continuous wavelet transform (CWT) was used for feature dimension reduction and extraction of hyperspectral wavelet features, while the Z-score algorithm was used to extract features for tobacco leaf area index and chlorophyll content. Finally, by comparing three machine learning classification algorithms—Random Forest, Support Vector Machine (SVM), and Backpropagation Neural Network (BPNN)—it was found that the Random Forest algorithm significantly outperforms the other two algorithms in both accuracy and Kappa value. The tobacco disease classification model based on multi-source remote sensing data and the Random Forest algorithm showed excellent performance in identifying tobacco disease categories, with an overall accuracy (OA) of 88.9%. Compared with the SVM and BPNN algorithms, the OA of this model improved by more than 14%.

We will focus on selecting appropriate deep learning models to analyze and classify large tobacco datasets in order to improve tobacco disease classification accuracy in future. First and foremost, we will prioritize the use of near-surface remote sensing imagery to comprehensively analyze the tobacco leaf canopy structure, texture, and related information, and obtain more diverse leaf samples representing different tobacco varieties as an independent dataset. Additionally, to enrich the multi-source features of tobacco leaves, we will integrate physicochemical data, such as the sodium, chloride, and potassium content of the leaves, as additional features to enhance the classification accuracy of tobacco.

These findings have practical implications for real-world agricultural management, enabling timely and accurate classification of tobacco diseases. The proposed framework can help farmers and agronomists implement targeted interventions, reduce yield losses, and improve overall crop quality.

## Data Availability

The raw data supporting the conclusions of this article will be made available by the authors, without undue reservation.

## References

[B1] Bao D. Zhou J. Bhuiyan S. A. Adhikari P. Tuxworth G. Ford R. . (2024). Early detection of sugarcane smut and mosaic diseases via hyperspectral imaging and spectral–spatial attention deep neural networks. J. Agric. Food Res. 18, 101369. doi: 10.1016/j.jafr.2024.101369

[B2] Boulent J. Foucher S. Théau J. St-Charles P.-L. (2019). Convolutional neural networks for the automatic identification of plant diseases. Front. Plant Sci. 10. doi: 10.3389/fpls.2019.00941, PMID: 31396250 PMC6664047

[B3] Breiman L. (2001). Random forests. Mach. Learn. 45, 5–32. doi: 10.1023/A:1010933404324

[B4] Chadoulis R. T. Livieratos I. Manakos I. Spanos T. Marouni Z. Kalogeropoulos C. . (2025). 3D-CNN detection of systemic symptoms induced by different Potexvirus infections in four Nicotiana benthamiana genotypes using leaf hyperspectral imaging. Plant Methods 21, 15. doi: 10.1186/s13007-025-01337-0, PMID: 39930522 PMC11809018

[B5] Chatzidimopoulos M. Tsiouni M. Lagoudas C. Loridas A. Baliktsis S. Vozikis C. (2024). “ Unmanned aerial systems for early detection of downy mildew in tobacco fields: enhancing financial outcomes through precision monitoring,” in Proc. Tenth Int. Conf. Remote Sens. Geoinformation Environ. (RSCy2024), Paphos, Cyprus ( SPIE). 384–387.

[B6] Chen H. Han Y. Liu Y. Liu D. Jiang L. Huang K. . (2023). Classification models for tobacco mosaic virus and potato virus Y using hyperspectral and machine learning techniques. Front. Plant Sci. 14. doi: 10.3389/fpls.2023.1211617, PMID: 37915507 PMC10617679

[B7] Cheng X. Feng Y. Guo A. Huang W. Cai Z. Dong Y. . (2023). Detection of rubber tree powdery mildew from leaf-level hyperspectral data using continuous wavelet transform and machine learning. Remote Sens. 16, 105. doi: 10.3390/rs16010105

[B8] Fan P. Ma C. Zhang L. Li J. Su Z. Li H. (2024). “ Research on detection method of moldy tobacco leaf raw materials based on hyperspectral and machine learning,” in In Proc. Int. Conf. Optics Machine Vision (ICOMV 2024), Xi’an, China ( SPIE). 308–314.

[B9] Fei R. Guo Y. Li J. Hu B. Yang L. (2023). An improved BPNN method based on probability density for indoor location. IEICE Trans. Inf. Syst. 106, 773–785. doi: 10.1587/transinf.2022DLP0073

[B10] Haagsma M. Hagerty C. H. Kroese D. R. Selker J. S. (2023). Detection of soil-borne wheat mosaic virus using hyperspectral imaging: from lab to field scans and from hyperspectral to multispectral data. Precis. Agric. 24, 1030–1048. doi: 10.1007/s11119-022-09986-0

[B11] Karatzoglou A. Meyer D. Hornik K. (2006). Support vector machines in R. J. Stat. Software 15, 1–28. doi: 10.18637/jss.v015.i09

[B12] Li J. Sun W. Liu S. Cheng T. Tang L. Jiang W. (2025). Prediction and mapping of tobacco yield with fresh leaf mass using hyperspectral sensing data. Smart Agric. Technol. 10, 100855. doi: 10.1016/j.atech.2025.100855

[B13] Lin J. Chen Y. Pan R. Cao T. Cai J. Yu D. (2022). CAMFFNet: a novel convolutional neural network model for tobacco disease image recognition. Comput. Electron. Agric. 202, 107390. doi: 10.1016/j.compag.2022.107390

[B14] Malvern Panalytical (2025). ASD portable spectrometers and spectroradiometers. Available online at: https://www.malvernpanalytical.com/en/products/product-range/asd-range (Accessed May 23, 2025).

[B15] Mao C. Zhao Y. Wang L. Yang Z. Weili K. Xu W. (2025). Machine learning-enabled UAV hyperspectral identification of tomato spotted wilt virus in tobacco. Front. Plant Sci. 16, 1728043. doi: 10.3389/fpls.2025.1728043, PMID: 41446678 PMC12722443

[B16] Mile People’s Government (2025). Overview of Mile City. Available online at: https://www.hhml.gov.cn/info/5141/647252.htm (Accessed May 23, 2025).

[B17] Reis Pereira M. Verrelst J. Tosin R. Rivera Caicedo J. P. Tavares F. Neves dos Santos F. (2024). Plant disease diagnosis based on hyperspectral sensing: comparative analysis of parametric spectral vegetation indices and nonparametric Gaussian process classification approaches. Agronomy 14, 493. doi: 10.3390/agronomy14030493

[B18] Sawyer E. Laroche-Pinel E. Flasco M. Cooper M. L. Corrales B. Fuchs M. (2023). Phenotyping grapevine red blotch virus and grapevine leafroll-associated viruses before and after symptom expression through machine-learning analysis of hyperspectral images. Front. Plant Sci. 14, 1117869. doi: 10.3389/fpls.2023.1117869, PMID: 36968421 PMC10036814

[B19] Sigit F. M. Syaifudin R. Suryaningrum D. A. (2022). “ Disease detection system in tobacco leaves based on edge detection with decision tree classification method,” in Proc. Int. Seminar Business Educ. Sci, Kediri, Indonesia: Universitas Nusantara PGRI Kediri. 224–232.

[B20] Smola A. J. Schölkopf B. (2004). A tutorial on support vector regression. Stat. Comput. 14, 199–222. doi: 10.1023/B:STCO.0000035301.49549.88

[B21] Tian C. Lu Y. Xie H. Yu Y. Lu L. (2025). Retrieval of nicotine content in cigar leaves by remote analysis of aerial hyperspectral combining machine learning methods. Sci. Rep. 15, 3895. doi: 10.1038/s41598-025-88091-4, PMID: 39890995 PMC11785768

[B22] Vapnik V. (1999). The Nature of Statistical Learning Theory (Berlin: Springer).

[B23] Wang Y. M. Ostendorf B. Pagay V. (2024a). Evaluating the potential of high‑resolution hyperspectral UAV imagery for grapevine viral disease detection in Australian vineyards. Int. J. Appl. Earth Obs. Geoinf. 130, 103876. doi: 10.1016/j.jag.2024.103876

[B24] Wang Y. M. Pagay V. (2024b). “ Rapid detection of grapevine viral disease with high-resolution hyperspectral remote sensing technology,” in Proc. IEEE International Geoscience and Remote Sensing Symposium (IGARSS 2024), Athens, Greece: IEEE. 4303–4306.

[B25] Wang S. Qin Y. Zhang F. Sun W. Lin X. Wu H. (2025). Retrieval of tobacco canopy chlorophyll content by integrating multispectral vegetation indices and texture features. Smart Agric. Technol. 12, 101268. doi: 10.1016/j.atech.2025.101268

[B26] Yang M. Kang X. Qiu X. Ma L. Ren H. Huang C. (2024). Method for early diagnosis of verticillium wilt in cotton based on chlorophyll fluorescence and hyperspectral technology. Comput. Electron. Agric. 216, 108497. doi: 10.1016/j.compag.2023.108497

[B27] Yin J. Wang J. Jiang J. Xu J. Zhao L. Hu A. (2024). Quality prediction of air-cured cigar tobacco leaf using region-based neural networks combined with visible and near-infrared hyperspectral imaging. Sci. Rep. 14, 31206. doi: 10.1038/s41598-024-82586-2, PMID: 39732746 PMC11682218

[B28] Zeng X. Li Y. Li J. Pu Z. Zheng L. Song P. (2023). Multi-sensor-based method for early detection of bacterial wilt of tobacco. Int. J. Precis. Agric. Aviat. 6, 33–43. doi: 10.33440/j.ijpaa.20230601.219

[B29] Zhang M. Chen T. E. Gu X. Chen D. Wang C. Wu W. (2023a). Hyperspectral remote sensing for tobacco quality estimation, yield prediction, and stress detection: a review of applications and methods. Front. Plant Sci. 14, 1073346. doi: 10.3389/fpls.2023.1073346, PMID: 36968402 PMC10030857

[B30] Zhang M. Chen T. E. Gu X. Kuai Y. Wang C. Chen D. (2023b). UAV-borne hyperspectral estimation of nitrogen content in tobacco leaves based on ensemble learning methods. Comput. Electron. Agric. 6(1), 33–43. doi: 10.1016/j.compag.2023.108008

[B31] Zhang X. Vinatzer B. A. Li S. (2024). Hyperspectral imaging analysis for early detection of tomato bacterial leaf spot disease. Sci. Rep. 14, 27666. doi: 10.1038/s41598-024-78650-6, PMID: 39532930 PMC11557939

[B32] Zhang B. Zhang M. Chen T. E. Chen D. Xu X. Yang X. (2026). Nitrogen application decision-making scheme for tobacco growth based on UAV multispectral imagery. J. Remote Sens. in press.

[B33] Zhang M. Zhang B. Zhao C. Chen L. Kuai Y. Wang C. (2025). Tobacco yield estimation via multi-source data fusion and recurrent neural networks. Int. J. Appl. Earth Obs. Geoinf. 144, 104925. doi: 10.1016/j.jag.2025.104925

[B34] Zhang B. Zhang M. Chen T. E. Chen D. Xu X. Yang X. . (2026). A nitrogen application decision-making scheme for tobacco growth based on UAV multispectral imagery. J. Remote Sens. 6, 0836. doi: 10.34133/remotesensing.0836

